# Who Reviews the Rewrite? Separating Patient-Initiated and Institutionally Curated Large Language Model Simplification

**DOI:** 10.2196/107277

**Published:** 2026-08-31

**Authors:** Aakash Dave

**Affiliations:** 1 Department of Psychology, Center for Bioethics and Social Justice, Institute for Quantitative Health Science and Engineering, Michigan State University, 775 Woodlot Dr, East Lansing, MI, United States, 1 5863565111

**Keywords:** health information, patient education material, readability, large language models, LLMs, AI

Miftaroski and colleagues [1] demonstrate that several large language models (LLMs) can reduce the linguistic complexity of German patient education materials; the interpretation of these findings is, however, complicated by whether the inquiry was patient initiated or institutionally curated.

The study approximates lay use by entering medical text into various publicly accessible LLMs with zero-shot prompts and no model parameter tuning. However, they conclude that rephrased material requires expert review to preserve medical accuracy and completeness. Thus, there are two distinct workflows that are not interchangeable. Expert review is a plausible safeguard when an institution revises material before publication, but it cannot be assumed when patients themselves seek an immediate explanation of information they do not understand.

This distinction is further complicated by the reliability analyses, in which 3 reviewers with medical informatics backgrounds achieved a Fleiss κ of 0.264% and an agreement of 54.6% when identifying false or decontextualized statements [1]. The authors attribute this low agreement, at least in part, to a lack of deep domain expertise, with the source being incompletely characterized. It seems that the panel had heterogeneous academic backgrounds, and the study fails to report important variables: prespecified rating rubric, reviewer calibration, domain-specific clinical expertise, reviewer-level ratings, or an adjudication process. The authors also probe accuracy, clarity, and plausibility, but it is unclear whether these were evaluated as distinct constructs or incorporated into a single judgment. I argue that the observed κ value cannot distinguish ambiguity within the outputs from heterogeneity in reviewer knowledge or decision thresholds, given that the Guidelines for Reporting Reliability and Agreement Studies highlight the importance of rater selection, training, and the measurement process [2].

These limitations are consequential, because readability and clinical adequacy are not the same. For example, Flesch Reading Ease and Wiener Sachtextformel scores quantify features of words and sentences [1], but they do not establish whether the patient is actually informed by or can act on the resultant information. Moreover, the Patient Education Materials Assessment Tool separately evaluates understandability and actionability [3], while direct testing can determine whether readers correctly interpret and apply health information [4]. Thus, improved readability scores do not directly support the inference that unreviewed LLM simplification is directly linked to improvement in patient understanding [3,4].

In future studies within this realm, the authors should prespecify the intended workflow. Materials that are intended for patients ideally should be assessed for all, if not some of the following: comprehension, error recognition, intended actions, trust calibration, and performance across health literacy levels. Next, institutionally generated patient education materials should also ascertain if domain experts can reliably identify clinically meaningful omissions or distortions and whether the additional review process actually provides a measurable advantage over conventional human authorship.

Given the aforementioned concerns, what we can conclude from this paper is that LLMs can alter linguistic complexity, but not that this translates to the production of clinically safe patient communication. In brief, medical text may become easier to read without becoming safer to understand per se.

## Abbreviations

LLM: large language model
